# Detection of *Porphyromonas gingivalis* and *Aggregatibacter actinomycetemcomitans* after Systemic Administration of Amoxicillin Plus Metronidazole as an Adjunct to Non-surgical Periodontal Therapy: A Systematic Review and Meta-Analysis

**DOI:** 10.3389/fmicb.2016.01277

**Published:** 2016-08-19

**Authors:** Aleksandar Dakic, Adrien Boillot, Cyrille Colliot, Maria-Clotilde Carra, Sébastien Czernichow, Philippe Bouchard

**Affiliations:** ^1^Department of Periodontology, Service of Odontology, U.F.R. of Odontology, Rothschild Hospital, AP-HP, Paris Diderot UniversityParis, France; ^2^UMS 011, Population-based Epidemiologic Cohorts Unit, Institut National de la Santé et de la Recherche MédicaleVillejuif, France; ^3^Department of Nutrition, Hôpital Européen Georges Pompidou, AP-HP, Paris Descartes UniversityParis, France; ^4^EA 2496, U.F.R. of Odontology, Paris Descartes UniversityMontrouge, France

**Keywords:** *Porphyromonas gingivalis*, *Aggregatibacter actinomycetemcomitans*, periodontitis/therapy, amoxicillin, metronidazole, meta-analysis

## Abstract

**Objective:** To evaluate the variations in the detection of *Porphyromonas gingivalis* and/or *Aggregatibacter actinomycetemcomitans* before and after systemic administration of amoxicillin plus metronidazole in association with non-surgical periodontal therapy (NSPT).

**Background:** The adjunctive use of antibiotics has been advocated to improve the clinical outcomes of NSPT. However, no systematic review has investigated the microbiological benefit of this combination.

**Materials and Methods:** An electronic search was conducted up to December 2015. Randomized clinical trials comparing the number of patients testing positive for *P. gingivalis* and/or *A. actinomycetemcomitans* before and after NSPT with (test group) or without (control group) amoxicillin plus metronidazole were included. The difference between groups in the variation of positive patients was calculated using the inverse variance method with a random effects model.

**Results:** The frequency of patients positive for *A. actinomycetemcomitans* was decreased by 30% (*p* = 0.002) and by 25% (*p* = 0.01) in the test group compared to the control group at 3- and 6-month follow-up, respectively. Similar findings were observed when considering the frequency of patients positive for *Porphyromonas gingivalis*, with a reduction by 28% (*p* < 0.0001), 32% (*p* < 0.0001), and 34% (*p* = 0.03) in the test group compared to the control group at 3-, 6-, and 12-month follow-up, respectively.

**Conclusion:** The systemic administration of amoxicillin plus metronidazole as an adjunct to NSPT significantly decreased the number of patients positive for *P. gingivalis* and *A. actinomycetemcomitans* compared with periodontal therapy alone or with a placebo.

## Introduction

Periodontitis is associated with an accumulation of microorganisms organized as a biofilm onto the dental root surface. The microbial composition of the dental biofilm includes a large number of oral bacteria, among which only a limited number of periodontal pathogens (Kolenbrander et al., 2010). Among these bacteria, *Porphyromonas gingivalis* (*P. gingivalis)* and *Aggregatibacter actinomycetemcomitans* (*A. actinomycetemcomitans)* are considered to be major periodontal pathogens (American Association of Periodontology, 1996)

Non-surgical periodontal therapy (NSPT) aims to reduce the supra- and sub-gingival microbial load by the mechanical disruption of the bacterial biofilm along the root surfaces (Darveau, 2010). NSPT is defined as the mechanical plaque removal, supra- and sub-gingival scaling, and root surface debridement. It is carried out using various types of instruments, such as hand instruments, sonic, and ultrasonic instruments. It can be conducted alone or with the adjunctive use of chemical antimicrobial agents. NSPT has been associated with the reduction of the main periodontal pathogens, including *P. gingivalis* and *A. actinomycetemcomitans* (Piconi et al., 2009).

Systemic administration of antibiotics has been advocated to improve the microbiological effects of NSPT (van Winkelhoff et al., 1996). In the last two decades, systematic reviews with meta-analysis compared the clinical effects of several combinations of antibiotics in complement to NSPT. A recent systematic review with meta-analysis indicated that the combination of amoxicillin (AMX) plus metronidazole (MTZ) was associated with an improvement of clinical periodontal variables, including probing pocket depth, and clinical attachment gain (Keestra et al., 2015a,b), Moreover, residual probing depth has been associated with the odds of detecting *P. gingivalis* (Mombelli et al., 2000). High levels of *P. gingivalis* and *A. actinomycetemcomitans* were also observed in non-responding sites (Fujise et al., 2002).

On the other hand, antimicrobial resistance threatens the effective prevention and treatment of an ever-increasing range of infections caused by bacteria (Anonymous, 2015). The administration of combined antibiotic therapy for Gram-negative bacteria remains controversial, and may increase the probability of resistance (van Winkelhoff et al., 2005; Feres et al., 2015). Thus, the risk-benefit ratio of the use of systemic AMX + MTZ in addition to NSPT in chronic periodontitis may be challenged. Indeed, the use of antibiotics does not belong to the standard treatment guidelines of chronic periodontitis (Herrera et al., 2002, 2008, 2012; Drisko, 2014). To date, no meta-analysis has evaluated the microbiological benefits of the combination of mechanical and antimicrobial therapies on two major periodontal pathogens; i.e., *P. gingivalis* and *A. actinomycetemcomitans*.

The aim of the present systematic review and meta-analysis is to evaluate the detection of *P. gingivalis* and/or *A. actinomycetemcomitans* after NSPT with or without systemic administration of AMX + MTZ.

## Materials and methods

### Data sources and literature search

A systematic review with meta-analysis was performed according to the Preferred Reporting Items for Systematic Reviews and Meta-Analysis (PRISMA) guidelines (Moher et al., 2015). Relevant articles published in the English language were identified up to December 2015, from MEDLINE, EMBASE, and Cochrane Library databases. Gray literature was also explored by searching non-published randomized controlled trials (RCTs) in ICTRP (WHO), OpenSIGLE, and ClinicalTrials.gov registers. Finally, electronic searches limited to abstracts were conducted in the main dental and periodontal journals (i.e., *Journal of Dental Research, Journal of Periodontology, Annals of Periodontology, Clinical Advances in Periodontics, Journal of Clinical Periodontology, Journal of Periodontal Research*, and *Oral Microbiology and Immunology*). The search strategies are detailed in Supplemental Table 1. The references listed of articles of interest and in the main systematic reviews on the topic were scrutinized to identify other relevant articles (Supplemental Table 2).

### Study selection

The selection included RCTs with (i) patients receiving systemic administration of AMX + MTZ as an adjunct to NSPT (test group) vs. patients receiving either NSPT alone or with a placebo (control group); (ii) microbiological technologies aiming to identify *P. gingivalis* and/or *A. actinomycetemcomitans* from subgingival plaque samples; (iii) a follow-up of at least 3 months; (iv) dichotomous data indicating the presence/absence of *P. gingivalis* and *A. actinomycetemcomitans*.

Exclusion criteria were the following: (i) studies published in non-English language, (ii) non-randomized trials, (iii) trials that included surgical periodontal therapy.

### Validity assessment

Study selection was carried out independently by two blind reviewers (CC and AD). The titles of articles retrieved from the electronic search were screened. The abstracts of relevant articles were examined, and all studies that could be included were retrieved. Discrepancies with regard to the inclusion or exclusion of studies were resolved by discussion between the reviewers (CC and AD). If a disagreement persisted, the judgment of a third reviewer (PB) was considered decisive. The kappa coefficient used to assess inter-rater reliability between the two reviewers was 0.91 for the global process of study selection (CI_95%_: 0.81–1.00).

### Study characteristics

The following data were extracted from each study in blinded conditions by two independent investigators (AD and AB): first author, year of publication, country, definition of cases, sample size with loss of follow-up, mean age, sampling strategy, microbiological technology, follow-up duration, type of treatment, antibiotic regimens, maintenance regimen, and main results.

The quality assessment of studies was evaluated in blinded conditions by two independent investigators (AD and AB) using the Cochrane Collaboration tool for assessment of the risk of bias. This tool assesses seven main criteria (sequence generation, allocation concealment, blinding of participants and staff, blinding of outcome examiners, incomplete outcome data, selective outcome reporting, and other sources of bias), all recorded as adequate, unclear, or inadequate. We considered studies were at low risk of bias if all criteria were met, and at high risk of bias if not.

### Data analysis

In order to identify possible unpublished data in the selected studies, the corresponding authors of the included articles were contacted by email. Because the number of patients positive for a bacterium could either increase or decrease during the follow-up compared to baseline, the outcome was treated as a continuous variable rather than a dichotomous variable. This variable was defined as a “variation of positive patients.” A patient was positive when the pathogen was detected. For test and control groups, the difference in the number of positive patients from baseline to follow-up examination was weighted. The following formula was used: *(a*−*b)/a*, where *a* is the number of positive patients at baseline and *b* is the number of positive patients at follow-up. The “variation of positive patients” was then calculated in the test and control groups.

The pooled difference for the “variation of positive patients” between the test and control groups was calculated using the inverse variance method. A random effects model was selected to take into account heterogeneity due to the low sample size of studies dealing with the topic. Subgroup analyses were constructed according to the protocol of NSPT (full-mouth disinfection vs. classical approach), sampling strategy (deepest sites sampling vs. sampling of sites with various probing depths), microbiological technology (low-sensitivity technologies vs. high-sensitivity technologies), and type of periodontitis (aggressive and chronic periodontitis). Only subgroups including two studies or more were analyzed. The statistical significance was set at *p* < 0.05, and 95% confidence interval (95%CI) was calculated. The percentage of variability across studies attributable to heterogeneity rather than chance was estimated using the *I*^2^ statistic (Higgins et al., 2003). All analyses were performed using R (R, version 3.2.3, R Development Core Team (2008), R Foundation for Statistical Computing, Vienna, Austria) and Review Manager (RevMan, version 5.2.8, The Cochrane Collaboration (2012), The Nordic Cochrane Centre, Copenhagen, Denmark).

## Results

### Literature search

**Figure 1:**
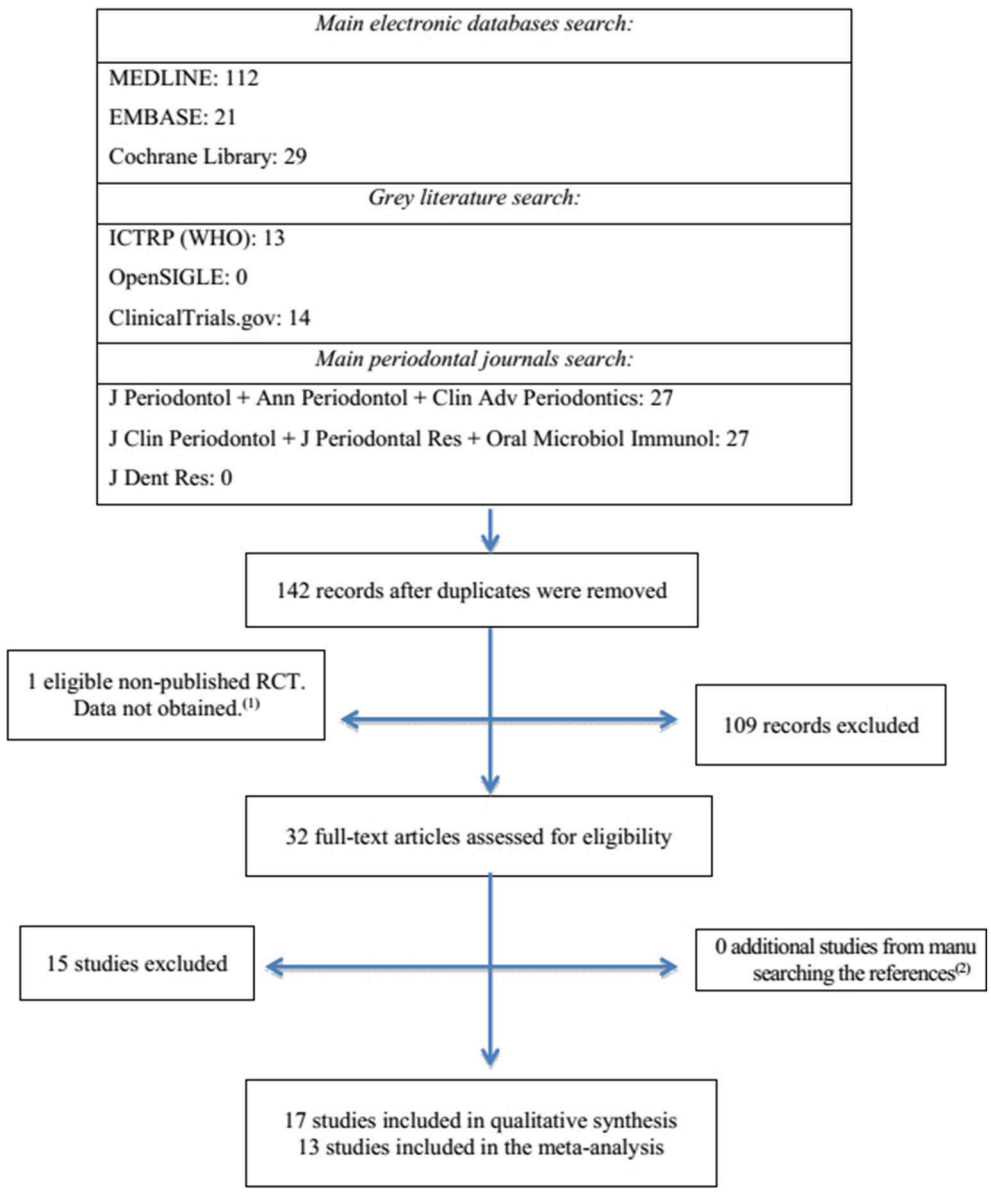
**Flowchart of study inclusion process**.

After removal of the duplicate articles, 142 studies were identified from the electronic search. Reading titles and abstracts reduced the number to a total of 32 eligible studies. One eligible non-published randomized clinical trial was identified in the gray literature (ICTRP database, Trial ID: ISRCTN17605083). The corresponding author was contacted but data were not obtained. After reading of the full articles, 15 studies were excluded. Reasons for exclusion are described in Supplemental Table 3. Finally, 13 studies were included in the meta-analysis: data of interest were found directly in seven studies (Winkel et al., 2001; Rooney et al., 2002; Ehmke et al., 2005; Cionca et al., 2010; Mestnik et al., 2010; Aimetti et al., 2012; Guerrero et al., 2014). Unpublished data were obtained from the authors in six studies (Xajigeorgiou et al., 2006; Matarazzo et al., 2008; Silva et al., 2011; Silva-Senem et al., 2013; Miranda et al., 2014; Soares et al., 2014; Figure 1).

### Description of included studies

Supplemental Table 4 summarizes the characteristics of the included studies. Five studies had a follow-up ≥12 month (Berglundh et al., 1998; Ehmke et al., 2005; Silva-Senem et al., 2013; Miranda et al., 2014; Soares et al., 2014). Full-mouth disinfection therapy was performed within 48 h or less in four studies (Ehmke et al., 2005; Cionca et al., 2010; Aimetti et al., 2012; Guerrero et al., 2014). The antibiotic regimens lasted between 7 and 14 days according to the different protocols used in the studies. Bacterial culture was used in three studies (Berglundh et al., 1998; Winkel et al., 2001; Rooney et al., 2002), PCR in eight studies (Ehmke et al., 2005; Ribeiro Edel et al., 2009; Cionca et al., 2010; Yek et al., 2010; Aimetti et al., 2012; Casarin et al., 2012; Guerrero et al., 2014; Miranda et al., 2014), and checkerboard DNA-DNA hybridization in six studies (Xajigeorgiou et al., 2006; Matarazzo et al., 2008; Mestnik et al., 2010; Silva et al., 2011; Silva-Senem et al., 2013; Soares et al., 2014).

The difference in the microbiological effect between groups was not available in four studies (Berglundh et al., 1998; Rooney et al., 2002; Matarazzo et al., 2008; Silva et al., 2011). In three studies, a higher reduction of both bacteria was observed in the test group compared with the control group (Cionca et al., 2010; Mestnik et al., 2010; Soares et al., 2014). The mean reduction of *A. actinomycetemcomitans* was founded to be higher in the test group than in the control group in two studies (Ehmke et al., 2005; Aimetti et al., 2012), and for *P. gingivalis* in two others (Guerrero et al., 2014; Miranda et al., 2014). This reduction was not significant between groups in six studies (Winkel et al., 2001; Xajigeorgiou et al., 2006; Ribeiro Edel et al., 2009; Yek et al., 2010; Casarin et al., 2012; Silva-Senem et al., 2013).

### Quantitative assessment

**Figure 2:**
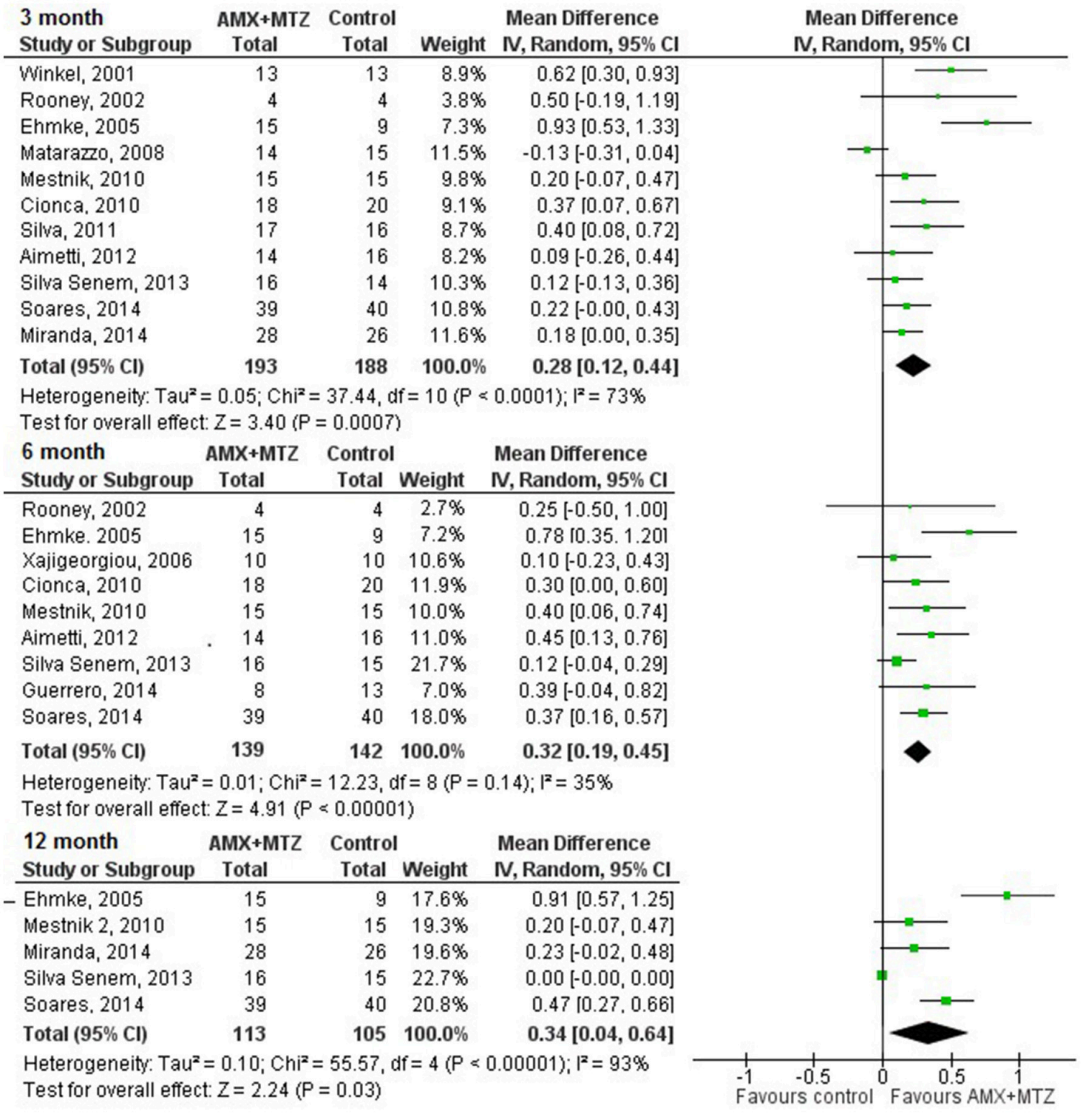
**Percentage of change in the detection of ***P. gingivalis*** at 3, 6, and 12 months (antimicrobial therapy in complement to NSPT (test) vs. NSPT alone or with placebo control)**.

In the pooled data analysis, the percentages of subjects positive for *P. gingivalis* at 3-, 6-, and 12-month follow-up in the test group compared with the control group were decreased by 28% (CI_95%_: 12–44), 32% (CI_95%_: 19–45), and 34% (CI_95%_: 4–64), respectively (Figure 2).

**Figure 3:**
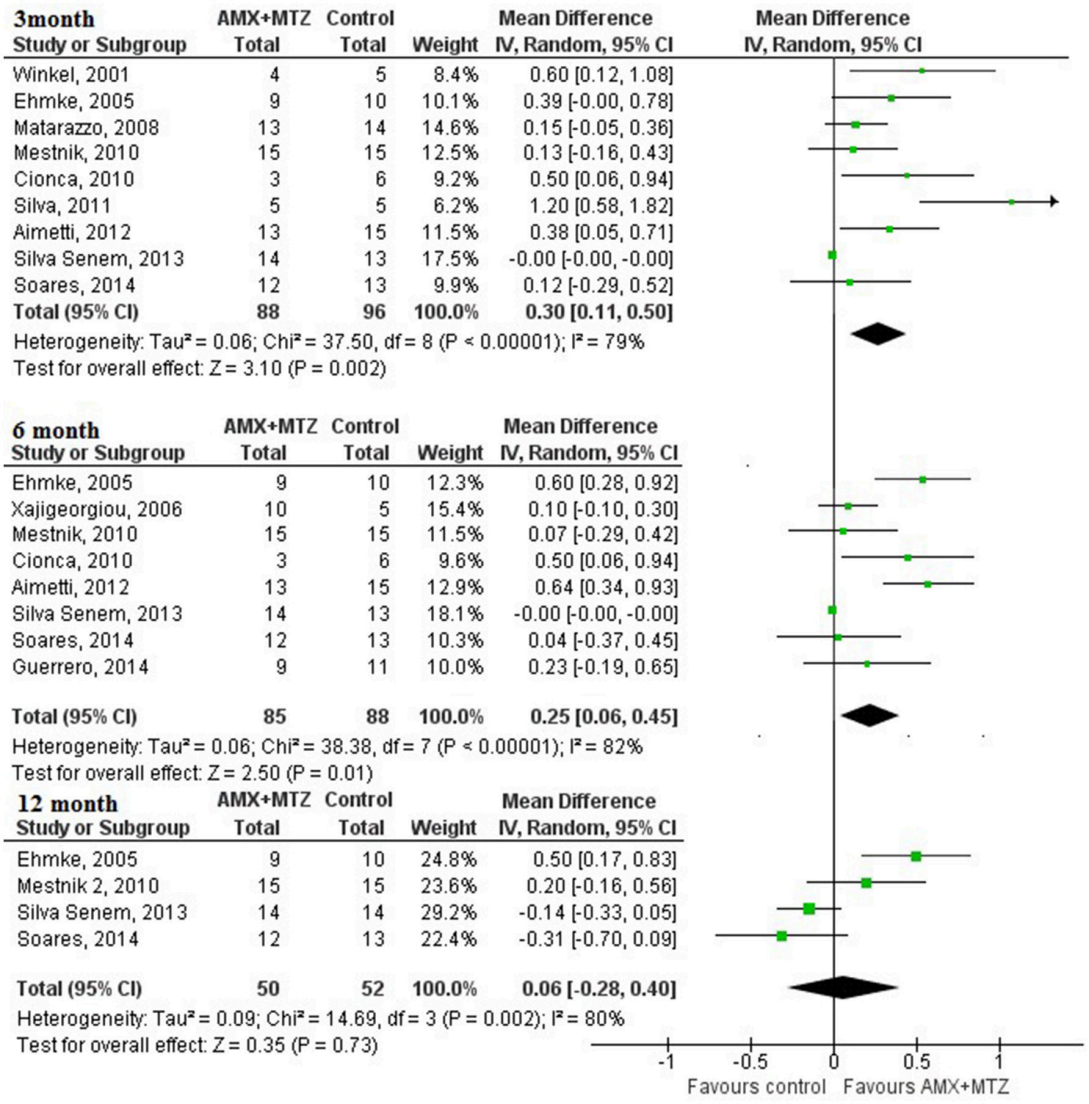
**Percentage of change in the detection of ***A. actinomycetemcomitans*** at 3, 6, and 12 months (antimicrobial therapy in complement to NSPT (test) vs. NSPT alone or with placebo control)**.

For the analysis of the variation of *A. actinomycetemcomitans, one* study was excluded from the meta-analysis because of a too small sample size (Rooney et al., 2002). At 3-month follow-up, the number of subjects positive for *A. actinomycetemcomitans* was decreased by 30% (CI_95%_: 11–50) in the test group compared with the control group. At 6-month follow-up, the number of subjects positive for *A. actinomycetemcomitans* was decreased by 25% (CI_95%_: 6–45) in the test group compared with the control group. At 12-month follow-up, no difference was observed between groups (Figure 3).

### Subgroup analyses

Subgroup analyses (Tables 1, 2 for 3- and 6-month follow-up; Supplemental Table 5 for 12-month follow-up) showed that the type of NSPT, the sampling strategy, or the microbiological technology did not influence the results for *P. gingivalis* at 3- and 6-month follow-up. The mean difference in the detection change of *A. actinomycetemcomitans* between groups was not significant when the classical NSPT was used (multiple session of NSPT), whatever the follow-up. This lack of significant change was also found when biofilm collection was done in sites with various probing depths at 6- and 12-month follow-up. The subgroup analyses dealing with the type of periodontitis indicated a significant difference between groups for both pathogens in chronic periodontitis. In contrast, no difference was found in aggressive periodontitis for both bacteria, expect for *P. gingivalis* at 6-month follow-up.

**Table 1:** **Impact of mechanical treatment protocol, sampling methods, microbiological technology, type of periodontitis and duration of antibiotic regimen on the percentage of change in the detection of ***A. actinomycetemcomitans*** at 3-, 6-, and 12-month follow-up**.

	**Mean difference (95% CI)**	***I*^2^(%)**
**AT 3-MONTH FOLLOW-UP**		
Full-mouth disinfection (1,2,3,8,10)	0.33 (0.04–0.63)^*^	80
Multiple session of NSPT (5,6,7,9)	0.30 (−0.02–0.62)	72
Biofilm collection, deep sites only (PD ≥ 6 mm) or deepest sites (2,3,10)	0.48 (0.23–0.73)^§^	0
Biofilm collection, sites with different probing depths (1,5,6,7,8,9)	0.22 (0.02–0.43)^*^	78
Microbiological technology, Non-enzymatic technique (1,2,3,5,6,7,8,9)	0.27 (0.08–0.47)^§^	78
Aggressive periodontitis (1,6,8)	0.12 (−0.09–0.34)	66
Chronic periodontitis (2,3,5,7,9,10)	0.42 (0.16–0.69)^§^	63
Antibiotic regimen for 7 days (1,2,10)	0.46 (0.23–0.70)^§^	0
Antibiotic regimen for 14 days (5,6,7,9)	0.30 (−0.02–0.62)	72
**AT 6-MONTH FOLLOW-UP**		
Full-mouth disinfection (1,2,3,4,8,11)	0.32 (0.07–0.57)^§^	87
Multiple session of NSPT (6,9)	−0.05 (−0.10–0.00)	0
Biofilm collection, deep sites only (PD ≥ 6 mm) or deepest sites (2,3,4,11)	0.34 (0.07–0.61)^*^	63
Biofilm collection, sites with different probing depths (1,6,8,9)	0.18 (−0.13–0.49)	83
Microbiological technology, Non-enzymatic technique (1,2,3,4,6,8,9,11)	0.25 (0.06–0.45)^*^	63
Aggressive periodontitis (1,4,6,8,11)	0.18 (−0.03–0.39)	80
Chronic periodontitis (2,3,9)	0.39 (0.05–0.73)^*^	57
Antibiotic regimen for 7 days (1,2,4,11)	0.35 (0.06–0.64)^*^	69
Antibiotic regimen for 14 days (6,9)	0.05 (−0.21–0.32)	0

*Inverse variance method, random effects model*.

* p < 0.05;

§ *p < 0.01. Full-mouth disinfection/multiple sessions: all non-surgical periodontal therapy (NSPT) realized within 48 h or less/in more than 48 h. PD, Probing depth. 1, Aimetti et al. (2012); 2, Cionca et al. (2010); 3, Ehmke et al. (2005); 4, Guerrero et al. (2014); 5, Matarazzo et al. (2008); 6, Mestnik et al. (2010); 7, Silva et al. (2011); 8, Silva-Senem et al. (2013); 9, Soares et al. (2014); 10, Winkel et al. (2001); 11, Xajigeorgiou et al. (2006)*.

**Table 2:** **Impact of mechanical treatment protocol, sampling methods, microbiological technology, type of periodontitis, and duration of antibiotic regimen on the percentage of change in the detection of ***P. gingivalis*** at 3- and 6-month follow-up**.

	**Mean difference (95% CI)**	***I*^2^(%)**
**AT 3-MONTH FOLLOW-UP**		
Full-mouth disinfection (1,2,3,10,12)	0.41 (0.12–0.70)^§^	76
Multiple session of NSPT (5,6,7,8,9,11)	0.17 (0.00–0.33)	62
Biofilm collection, deep sites only (PD ≥ 6 mm) or deepest sites (2,3,8,12)	0.60 (0.35–0.85)^§^	39
Biofilm collection, sites with different probing depths (1,5,6,7,9,10,11)	0.13 (0.01–0.26)^*^	51
Microbiological technology, Non-enzymatic technique (1,2,3,5,6,7,9,10,11)	0.23 (0.07–0.39)^§^	73
Microbiological technology, Enzymatic technique (8,12)	0.60 (0.31–0.88)^§^	0
Chronic periodontitis (2,3,5,7,8)	0.32 (0.00–0.65)^*^	85
Aggressive periodontitis (1,6,10)	0.14 (−0.02–0.30)	0
Antibiotic regimen for 7 days (1,2,8,12)	0.38 (0.14–0.62)^§^	40
Antibiotic regimen for 14 days (5,6,9,11)	0.15 (−0.08–0.38)	74
**AT 6-MONTH FOLLOW-UP**		
Full-mouth disinfection (1,2,3,4,10,13)	0.32 (0.13–0.50)^§^	54
Multiple session of NSPT (6,8,11)	0.37 (0.20–0.54)^§^	0
Biofilm collection, deep sites only (PD ≥ 6 mm) or deepest sites (2,3,4,8,13)	0.35 (0.12–0.58)^§^	37
Biofilm collection, sites with different probing depths (1,6,10,11)	0.30 (0.14–0.46)^§^	45
Microbiological technology, Non-enzymatic technique (1,2,3,4,6,10,11,13)	0.32 (0.19–0.46)^§^	43
Aggressive periodontitis (1,4,6,10,13)	0.25 (0.10–0.40)^§^	26
Chronic periodontitis (2,3,8,11)	0.41 (0.22–0.60)^§^	20
Antibiotic regimen for 7 days (1,2,4,8,13)	0.30 (0.14–0.46)^§^	0
Antibiotic regimen for 14 days (6,11)	0.38 (0.20–0.55)^§^	%

*Inverse variance method, random effects model*.

* p < 0.05;

§ *p < 0.01. Full-mouth disinfection/multiple sessions: all non-surgical periodontal therapy (NSPT) realized within 48 h or less/in more than 48 h. PD, Probing depth. 1, Aimetti et al. (2012); 2, Cionca et al. (2010); 3, Ehmke et al. (2005); 4, Guerrero et al. (2014); 5, Matarazzo et al. (2008); 6, Mestnik et al. (2010); 7, Miranda et al. (2014); 8, Rooney et al. (2002); 9, Silva et al. (2011); 10, Silva-Senem et al. (2013); 11, Soares et al. (2014); 12, Winkel et al. (2001); 13, Xajigeorgiou et al. (2006)*.

Only five studies met all criteria for low risk of bias (Mestnik et al., 2010; Aimetti et al., 2012; Casarin et al., 2012; Guerrero et al., 2014; Miranda et al., 2014; Supplemental Figure 1). When limiting the analyses to those studies, results were unchanged, except for the mean difference in the detection change of *A. actinomycetemcomitans* at 6-month follow-up, which was not significant (for *P. gingivalis/A. actinomycetemcomitans* at 3- and 6-month follow-up, respectively 17% (CI_95%_: 4–30)/24% (CI_95%_: 0–48) and 42% (CI_95%_: 21–62)/33% (CI_95%_: −4–69)).

## Discussion

The results of the present meta-analyses show that at 6-month follow-up the number of positive subjects for *P. gingivalis* is reduced by 32% (*p* < 0.001) in the test group treated by NSPT in adjunction of systemic antibiotics compared to control. At 6-month follow-up, the number of positive subjects for *A. actinomycetemcomitans* was reduced by 25% (*p* = 0.01) in the test group compared to control. At 12-month follow-up, a significant reduction between groups was still observed for *P. gingivalis;* whereas, this difference was not significant for *A. actinomycetemcomitans*. This lack of significance could be related to the few data available at more than 6-month of follow-up, and, as a consequence, it should be interpreted with caution. In the same vein, few studies are available for the performed subgroup analyses; particularly, the analyses limited to the studies assessed at low risk of bias resulted in questionable results. For instance, it failed to show a significant difference between groups for the detection of *A. actinomycetemcomitans* when classical NSPT was used (multiple session of NSPT). When a full-mouth disinfection protocol was implemented, this difference was significant (*p* < 0.01) (Table 1). This result may strengthen the fact that NSPT must be carried out in the shortest possible time span in case of patients positive for *A. actinomycetemcomitans* and for who antibiotic therapy is planned (Herrera et al., 2012). Similarly, whereas a significant difference was observed between groups when antibiotics were taken for 7 days, no significant difference was observed for a longer duration. Included studies in which antibiotics were taken for 7 days were all conducted with a full-mouth disinfection protocol, whereas multiple session of NSPT were used in studies with longer antimicrobial treatments. Despite the beneficial effect of adjunctive antimicrobial regimen compared with control, this outcome indicates that mechanical debridement is the cornerstone of periodontal therapy.

Interestingly, the adjunction of AMX + MTZ to NSPT does not bring advantages regarding *A. actinomycetemcomitans* in aggressive periodontitis. Several studies have demonstrated that the detection and quantity of *A. actinomycetemcomitans* is higher in patients with this type of periodontitis (Könönen and Müller, 2014). Thus, on a biological point of view, our results showing a lack of statistically significant difference between the test and control groups in aggressive periodontitis might be explained by the difficulty in reaching a level of non-detection for *A. actinomycetemcomitans* in this specific type of patients, even when antibiotics are used. Moreover, on a methodological point of view, the dichotomic approach using the patient as the statistical unit may also contribute to explain the absence of significant difference between groups. Indeed, it is possible that the mean quantity of *A. actinomycetemcomitans* was significantly reduced after treatment but remains above the detection threshold also in the test group (Table 1).

Six studies included in the quantitative analyses, and sharing similar clinical data, allowed to further explore the relationship between improved clinical parameters (in full-mouth, moderate and severe pockets) and variations in the number of positive subjects for both bacteria (Winkel et al., 2001; Matarazzo et al., 2008; Mestnik et al., 2010; Silva et al., 2011; Aimetti et al., 2012; Miranda et al., 2014). Distributions of microbial and clinical parameters shared similar patterns, with better clinical results related to higher reduction in the number of positive subjects (data not shown).

The present systematic review and meta-analysis has several strengths. To our knowledge, this is the first meta-analysis to provide a quantitative evaluation of the microbiological effects of systemic administration of AMX + MTZ in adjunction to the standard NSPT. The major novelty of the present study is to compare the presence/absence of bacteria according to the detection threshold, instead of mean counts. The reduction of the mean count of bacteria is less clinically meaningful than the rate of non-detection. The non-detection approach guaranties levels compatible with periodontal health independently of baseline values. Also, a strict study selection was performed. Studies with less than 3-month follow-up were excluded because previous observations have found that most clinical and microbiological improvements were observed 3 months after NSPT and antimicrobial therapy (Badersten et al., 1984; Berglundh et al., 1998; Winkel et al., 2001; Haffajee et al., 2006; Cionca et al., 2010). Moreover, unpublished data were collected and gray literature was explored. Therefore, the sample size of the meta-analysis resulted relatively large (536 subjects at baseline; 13 RCTs). In addition, the 12-month follow-up strengthened the outcomes of RCTs, showing a tendency of bacterial recolonization over time.

However, the present study has also some limitations. Moderate to high heterogeneity was found, probably due to the differences between the included studies. The use of different microbiological technologies may also be challenged. Unfortunately, thresholds of detection were not indicated in all studies. Thus, the subgroup analysis with specific detection thresholds was not possible. Therefore, it could be raised that the outcomes are influenced by the sensitivity of microbiological technologies presenting different levels of detection. In the literature, it has been shown better detection properties for PCR and DNA-DNA hybridization compared with cultures and enzymatic techniques. Nevertheless, comparative studies have demonstrated a good to excellent agreement between quantitative PCR and culture when P. *gingivalis* and *A. actinomycetemcomitans* were investigated (Lau et al., 2004; Jervoe-Storm et al., 2005). In the same vein, when comparing PCR with checkerboard DNA hybridization, the agreement between the two technologies was good for *P. gingivalis* and *A. Actinomycetemcomitans* detection (Haffajee et al., 2009). Finally, detection thresholds may differ between two studies using the same microbiological technology. Therefore, we also conducted subgroup analyses based on microbiological technologies. High-detection technologies (PCR and DNA-DNA hybridization) were compared with low-detection technologies (cultures and enzymatic methods). These analyses did not change the main outcome (Tables 1, 2). It may also be assumed that patient's compliance may impact the results. Unfortunately, it was not possible to evaluate the influence of the periodontal maintenance regimen on bacterial recolonization, because maintenance programs were unclear in 8 out of the 13 included studies. Moreover, age and gender may also influence the results. In all studies included in the quantitative assessment, excepted one (Rooney et al., 2002), age, and gender did not differ between groups at baseline. When this latter study was removed, the conclusions were unchanged. The percentages of subjects positive for *P. gingivalis* at 3- and 6-month follow-up in the test group compared with the control group were decreased respectively by 27% (CI_95%_: 10–43) and 32% (CI_95%_: 19–46) (all *p* < 0.01). Finally, only *P. gingivalis* and *A. actinomycetemcomitans* were considered in this meta-analysis. Recent data indicate that *P. gingivalis* may serve as surrogate markers of the bacterial community dysbiosis (Hajishengallis et al., 2012). In addition, *P. gingivalis* is implicated, via gingipains, in the association between periodontal diseases and systemic disorders (Olsen and Potempa, 2014; Alfakry et al., 2016). *A. actinomycetemcomitans* has long been associated with aggressive periodontitis (Könönen and Müller, 2014; Herbert et al., 2016). In addition, *A. actinomycetemcomitans* may play a role in the polymicrobial synergy that initiate the disease (Nibali, 2015). Consequently, these bacteria may be view as major microbiological risk factors. This is the reason why there are numerous RCTs investigating these bacteria.

The systemic administration of amoxicillin plus metronidazole as an adjunct to NSPT significantly, but modestly, decreased the number of patients positive for *P. gingivalis* and *A. actinomycetemcomitans* compared with periodontal therapy alone or with a placebo.

## Author contributions

Substantial contributions to the conception or design of the work; or the acquisition, analysis, or interpretation of data for the work: AB, AD, CC, MC, SC, PB. Drafting the work or revising it critically for important intellectual content: AB, AD, CC, MC, SC, PB. Final approval of the version to be published: AB, AD, CC, MC, SC, PB. Agreement to be accountable for all aspects of the work in ensuring that questions related to the accuracy or integrity of any part of the work are appropriately investigated and resolved: AB, AD, CC, MC, SC, PB.

### Conflict of interest statement

The authors declare that the research was conducted in the absence of any commercial or financial relationships that could be construed as a potential conflict of interest.

## Supplementary material

Supplementary Table 1 **Search strategies according to electronic source**.

Supplementary Table 2 **Major reviews on the topic selected for manual reference searches**.

Supplementary Table 3 **Excluded studies and reasons for exclusion**.

Supplementary Table 4 **Description of studies included in the systematic review**.

Supplementary Table 5 **Impact of mechanical treatment protocol, sampling methods, microbiological technology and type of periodontitis on the percentage of change in the detection of P. gingivalis and A. actinomycetemcomitans at 12-month follow-up**.

Supplementary Figure 1 **Risk of bias assessment of included studies**.
