# Supplementary material for: Detection of Porphyromonas gingivalis and Aggregatibacter actinomycetemcomitans after Systemic Administration of Amoxicillin Plus Metronidazole as an Adjunct to Non-surgical Periodontal Therapy: A Systematic Review and Meta-Analysis
Source: Front Microbiol. 2016 Aug 19;7:1277. doi: 10.3389/fmicb.2016.01277 (PMC4990718; doi:10.3389/fmicb.2016.01277)
Supplement: Supplementary Table 1 — Search strategies according to electronic source. [file Table1.DOC]

*Supplemental Table 1.* Search strategies according to electronic source

| ***Source*** | ***Search Strategy*** | ***Limit(s)*** |
| --- | --- | --- |
| MEDLINE | (((((((((((((dental scaling[MeSH Terms]) OR dental scaling[Title/Abstract])) OR ((periodontal debridement[MeSH Terms]) OR periodontal debridement [Title/Abstract])) OR Periodontitis/therapy [Mesh]) OR "scaling" [TIAB]) OR "root planing" [TIAB]) OR "periodontal treatment" [TIAB]) OR "periodontal therapy" [TIAB])) AND ((Metronidazole[Mesh] OR Metronidazole[TIAB]) AND (Amoxicillin[Mesh] OR Amoxicillin[TIAB]))) AND (microbiol*[TIAB] OR (Porphyromonas gingivalis[Mesh] OR Porphyromonas gingivalis[TIAB]) OR (Actinobacillus actinomycetemcomitans[Mesh] OR Actinobacillus actinomycetemcomitans[TIAB] OR Aggregatibacter actinomycetemcomitans[TIAB])))) | English |
| EMBASE | preventive dentistry AND (metronidazole AND amoxicillin) AND (“porphyromonas gingivalis” OR “aggregatibacter actinomycetemcomitans” OR “actinobacillus actinomycetemcomitans”) | English;  only EMBASE |
| *Supplemental Table 1. (continued)* | | |
| ***Source*** | ***Search Strategy*** | ***Limit(s)*** |
| Cochrane Library | (amoxicillin AND metronidazole) AND (Porphyromonas gingivalis OR Actinobacillus actinomycetemcomitans OR Actinobacillus actinomycetemcomitans OR Aggregatibacter actinomycetemcomitans) | Trials and Reviews |
| ICTRP (WHO) | (amoxicillin AND metronidazole) AND periodontal | - |
| OpenSIGLE | (amoxicillin AND metronidazole) AND periodontal | - |
| ClinicalTrials.gov | (amoxicillin AND metronidazole) AND periodontal | - |
| J Dent Res | (amoxicillin AND metronidazole) AND (porphyromonas OR actinobacillus OR aggregatibacter) | Abstracts |
| J Periodontol  Ann Periodontol  Clin Adv Periodontics | (amoxicillin AND metronidazole) AND (porphyromonas OR actinobacillus OR aggregatibacter) | Abstracts |
| J Clin Periodontol  J Periodontal Res  Oral Microbiol Immunol | (amoxicillin AND metronidazole) AND (porphyromonas OR actinobacillus OR aggregatibacter) | Abstracts |
