# Supplementary material for: Detection of Porphyromonas gingivalis and Aggregatibacter actinomycetemcomitans after Systemic Administration of Amoxicillin Plus Metronidazole as an Adjunct to Non-surgical Periodontal Therapy: A Systematic Review and Meta-Analysis
Source: Front Microbiol. 2016 Aug 19;7:1277. doi: 10.3389/fmicb.2016.01277 (PMC4990718; doi:10.3389/fmicb.2016.01277)
Supplement: Supplementary Table 2 — Major reviews on the topic selected for manual reference searches. [file Table2.DOC]

*Supplemental Table 2.* Major reviews on the topic selected for manual reference searches

| Feres M, Figueiredo LC, Soares GMS, Faveri M. Systemic antibiotics in the treatment of periodontitis. *Periodontol 2000* 2015: **67**: 131–186.  Haffajee AD, Socransky SS, Gunsolley JC. Systemic anti-infective periodontal therapy. A systematic review. *Ann Periodontol* 2003: **8**: 115–181.  Herrera D, Alonso B, León R, Roldán S, Sanz M. Antimicrobial therapy in periodontitis: the use of systemic antimicrobials against the subgingival biofilm. *J Clin Periodontol* 2008: **35**: 45–66.  Herrera D, Matesanz P, Bascones-Martínez A, Sanz M. Local and systemic antimicrobial therapy in periodontics. *J Evid Based Dent Pract* 2012: **12**: 50–60.  Herrera D, Sanz M, Jepsen S, Needleman I, Roldán S. A systematic review on the effect of systemic antimicrobials as an adjunct to scaling and root planing in periodontitis patients. *J Clin Periodontol* 2002: **29 Suppl 3**: 136–159; discussion 160–162.  Keestra JA, Grosjean I, Coucke W, Quirynen M, Teughels W. Non-surgical periodontal  therapy with systemic antibiotics in patients with untreated aggressive periodontitis: a  systematic review and meta analysis*.* *J Periodontal Res* 2015; **50**: 689-706.  Keestra JA, Grosjean I, Coucke W, Quirynen M, Teughels W. Non-surgical periodontal  therapy with systemic antibiotics in patients with untreated chronic periodontitis: a  systematic review and meta analysis*.* *J Periodontal Res* 2015; **50**: 294-314.  Sgolastra F, Gatto R, Petrucci A, Monaco A. Effectiveness of systemic amoxicillin/metronidazole as adjunctive therapy to scaling and root planing in the treatment of chronic periodontitis: a systematic review and meta-analysis. *J Periodontol* 2012: **83**: 1257–1269.  Sgolastra F, Petrucci A, Gatto R, Monaco A. Effectiveness of systemic amoxicillin/metronidazole as an adjunctive therapy to full-mouth scaling and root planing in the treatment of aggressive periodontitis: a systematic review and meta-analysis. *J Periodontol* 2012: **83**: 731–743.  Teles RP, Haffajee AD, Socransky SS. Microbiological goals of periodontal therapy. *Periodontol 2000* 2006: **42**: 180–218.  Zandbergen D, Slot DE, Cobb CM, Van der Weijden FA. The clinical effect of scaling and root planing and the concomitant administration of systemic amoxicillin and metronidazole: a systematic review. *J Periodontol* 2013: **84**: 332–351. |
| --- |
