# Supplementary material for: Detection of Porphyromonas gingivalis and Aggregatibacter actinomycetemcomitans after Systemic Administration of Amoxicillin Plus Metronidazole as an Adjunct to Non-surgical Periodontal Therapy: A Systematic Review and Meta-Analysis
Source: Front Microbiol. 2016 Aug 19;7:1277. doi: 10.3389/fmicb.2016.01277 (PMC4990718; doi:10.3389/fmicb.2016.01277)
Supplement: Supplementary Table 3 — Excluded studies and reasons for exclusion. [file Table3.DOC]

*Supplemental Table 3.* Excluded studies and reasons for exclusion.

| ***Publication*** | ***Reasons for exclusion*** |
| --- | --- |
| Buchmann, R., G. Conrads, and A. Sculean, *Short-term effects of systemic antibiotics during periodontal healing.* Quintessence international, 2010. 41(4): p. 303-12. | Follow-up shorter than 3 months |
| Ehmke, B., et al., *Clonal infection with Actinobacillus actinomycetemcomitans following periodontal therapy.* Journal of dental research, 1999. 78(9): p. 1518-24. | Duplicate with Ehmke et al. (2005) |
| Ehmke, B., et al., *Multifactorial assessment of predictors for prevention of periodontal disease progression.* Clinical oral investigations, 2003. 7(4): p. 217-21. | Duplicate with Ehmke et al. (2005) |
| Faveri, M., et al., *Clinical and microbiologic effects of adjunctive metronidazole plus amoxicillin in the treatment of generalized chronic periodontitis: smokers versus non-smokers.* J Periodontol. 2014. 85(4): p.581-91. | No control group |
| Flemmig, T.F., et al., *Differential clinical treatment outcome after systemic metronidazole and amoxicillin in patients harboring Actinobacillus actinomycetemcomitans and/or Porphyromonas gingivalis.* Journal of clinical periodontology, 1998. 25(5): p. 380-7. | Duplicate with Ehmke et al. (2005) |

| *Supplemental Table 3.* Excluded studies and reasons for exclusion *(continued).* | |
| --- | --- |
| ***Publication*** | ***Reasons for exclusion*** |
| Flemmig, T.F., et al., *Differential effects of systemic metronidazole and amoxicillin on Actinobacillus actinomycetemcomitans and Porphyromonas gingivalis in intraoral habitats.* Journal of clinical periodontology, 1998. 25(1): p. 1-10. | Duplicate with Ehmke et al. (2005) |
| Heller, D., et al., *Impact of systemic antimicrobials combined with anti-infective mechanical debridement on the microbiota of generalized aggressive periodontitis: a 6-month RCT.* Journal of clinical periodontology, 2011. 38(4): p. 355-64. | Duplicate with Silva-Senem et al. (2013) |
| Lopez, N.J. and J.A. Gamonal, *Effects of metronidazole plus amoxicillin in progressive untreated adult periodontitis: results of a single 1-week course after 2 and 4 months.* Journal of periodontology, 1998. 69(11): p. 1291-8. | No patient received periodontal therapy |
| Lopez, N.J., et al., *Effects of metronidazole plus amoxicillin as the only therapy on the microbiological and clinical parameters of untreated chronic periodontitis.* Journal of clinical periodontology, 2006. 33(9): p. 648-60. | No patient received periodontal therapy |

*Supplemental Table 3.* *Excluded studies and reasons for exclusion (continued).*

| ***Publication*** | ***Reasons for exclusion*** | |
| --- | --- | --- |
| Mdala, I., et al., *Multilevel analysis of bacterial counts from chronic periodontitis after root planing/scaling, surgery, and systemic and local antibiotics: 2-year results.* Journal of oral microbiology, 2013. 5. | No specific data for *Aa* and *Pg* |  |
| Moeintaghavi, A., et al., *Adjunctive effects of systemic amoxicillin plus metronidazole with scaling and root planing: a randomized, placebo controlled clinical trial.* The journal of contemporary dental practice, 2007. 8(5): p. 51-9. | Follow-up shorter than 3 months |  |
| Mombelli, A., et al., *Are there specific benefits of amoxicillin plus metronidazole in Aggregatibacter actinomycetemcomitans-associated periodontitis? Double-masked, randomized clinical trial of efficacy and safety.* Journal of periodontology, 2013. 84(6): p. 715-24. | No microbiological data |  |
| Sigusch, B., et al., *A 2-step non-surgical procedure and systemic antibiotics in the treatment of rapidly progressive periodontitis.* Journal of periodontology, 2001. 72(3): p. 275-83. | No combination of amoxicillin plus metronidazole |  |
| Tinoco, E.M., et al., *Clinical and microbiological effects of adjunctive antibiotics in treatment of localized juvenile periodontitis. A controlled clinical trial.* Journal of periodontology, 1998. 69(12): p. 1355-63. | Some patients with surgical periodontal therapy |  |
| Winkel, E.G., A.J. van Winkelhoff, and U. van der Velden, *Additional clinical and microbiological effects of amoxicillin and metronidazole after initial periodontal therapy.* Journal of clinical periodontology, 1998. 25(11 Pt 1): p. 857-64. | Not an RCT | |
