# Supplementary material for: Detection of Porphyromonas gingivalis and Aggregatibacter actinomycetemcomitans after Systemic Administration of Amoxicillin Plus Metronidazole as an Adjunct to Non-surgical Periodontal Therapy: A Systematic Review and Meta-Analysis
Source: Front Microbiol. 2016 Aug 19;7:1277. doi: 10.3389/fmicb.2016.01277 (PMC4990718; doi:10.3389/fmicb.2016.01277)
Supplement: Supplementary Table 4 — Description of studies included in the systematic review. [file Table4.DOC]

*Supplemental Table 4.* Description of studies included in the systematic review.

| **Authors (year)**  **Country**  **Follow-up (Months)** | **Type of periodontitis & definition** | **Sampling strategy**  **Detection method** | **Groups: Sample size at baseline  (drop outs); Age** | **Type of treatment** | **Antibiotic regimen, duration & timing of initiation** | **Maintenance regimen** | **Main results** |
| --- | --- | --- | --- | --- | --- | --- | --- |
| Aimetti et al.  (2012)[[1]](#endnote-2)  Italy  6 months | Generalized aggressive periodontitis.  >2 sites with CAL & PD ≥6mm. | 4 randomly selected sites including  2 sites with PD between 4 and 5mm and  2 sites with PD≥6mm.  PCR. | Test:  19 (0); 36.3±3.2  Control:  20 (0); 35.7±2.8 | Test:  NSPT48+AMX+MTZ+chx  Control:  NSPT48+placebo+chx | AMX 500 mg t.i.d.  MTZ 500 mg t.i.d.  7 days.  Immediately after first session of NSPT. | Recalls after 2, 4, and 6 weeks, and after 2, 4 and 6 months. | Better non-detection in the test group, except for *Pg* at 3 months. Tendency towards recolonization. |
| Berglundh et al.  (1998)  Sweden  12 months | Unclear | The 3 deepest sites in each quadrant.  Culture, enzymatic. | Test:  8 (0)  Control:  8 (0)  Age (Min-Max) for all participants:  35-58 | Test:  NSPT+AMX+MTZ  Control:  NSPT+placebo | AMX 375 mg b.i.d.  MTZ 250 mg t.i.d.  14 days.  Immediately after NSPT. | Unclear | . No information about significant differences from baseline and between groups. Slight tendency towards recolonization for *Pg* in control group at 12 months. |

*NSPT48: non-surgical periodontal therapy realized within 48 hours or less, except ultrasonic debridement only (USd); NSPT: non-surgical periodontal therapy realized in more than 48 hours; USd: 45 minutes ultrasonic debridement in one session; AMX: amoxicillin; MTZ: metronidazole; chx: chlorhexidine; t.i.d.: three times per day; b.i.d.: two times per day; Aa: Aggregatibacter actinomycetemcomitans; Pg: Porphyromonas gingivalis.; *: included in the meta-analysis*

*Supplemental Table 4.* Description of studies included in the systematic review *(Continued)*.

| **Authors (year)**  **Country**  **Follow-up (Months)** | **Type of periodontitis & definition** | **Sampling strategy**  **Detection method** | **Groups: Sample size at baseline  (drop outs); Age** | **Type of treatment** | **Antibiotic regimen, duration & timing of initiation** | **Maintenance regimen** | **Main results** |
| --- | --- | --- | --- | --- | --- | --- | --- |
| Cionca et al.  (2010)*  Switzerland  6 months | Moderate to advanced chronic periodontitis.  ≥4 teeth with ≥1 site(s) with PD >4mm, CAL ≥2mm, and bone loss. | Deepest site in each quadrant.  PCR. | Test:  25 (2); 50.6±8.6  Control:  26 (2); 50.5±13.6 | Test:  NSPT48+AMX+MTZ+chx  Control:  NSPT48+placebo+chx | AMX 375 mg t.i.d.  MTZ 500 mg t.i.d.  7 days.  Immediately after NSPT. | Recalls after 3 and 6 months. | Better reduction for *Aa* and *Pg* at 3 months in the test group.  Tendency towards recolonization for *Pg* at 6 months. |
| Del Peloso Ribeiro et al.  (2009)  Brazil  6 months | Severe chronic periodontitis  ≥8 teeth with ≥1 site(s) with PD≥5mm, 2 of the eight teeth having PD≥7mm, and 2 sites with PD≥6mm. Presence of sites with CAL ≥5mm plus BOP | 1 site with PD ≥5mm  and 1 site with PD ≥7mm.  PCR. | Test:  12 (0); 46.0 (34-55)  Control:  13 (0); 46.2 (30-66) | Test:  USd+AMX+MTZ  Control:  USd+placebo | AMX 375 mg t.i.d.  MTZ 250 mg t.i.d.  7 days.  Immediately after NSPT. | Recalls every month for 6 months. | No difference for *Aa* and *Pg* reduction between groups. |
| Ehmke et al.  (2005)*  Germany  24 months | Moderate and severe chronic periodontitis.  Unclear definition. | Deepest site in each quadrant with PD ≥6mm.  PCR. | Test:  18 (0); 48.9±11·0  Control:  17 (0); 53.2±9·9 | Test:  NSPT48+AMX+MTZ+chx  Control:  NSPT48 | AMX 375 mg t.i.d.  MTZ 250 mg t.i.d.  8 days.  Immediately after NSPT. | Recalls at 3, 6, 9, 12, 18, and 24 months. | Better *Aa* non-detection in test group until 18 months.  No difference between groups for *Pg.* |

*NSPT48: non-surgical periodontal therapy realized within 48 hours or less, except ultrasonic debridement only (USd); NSPT: non-surgical periodontal therapy realized in more than 48 hours; USd: 45 minutes ultrasonic debridement in one session; AMX: amoxicillin; MTZ: metronidazole; chx: chlorhexidine; t.i.d.: three times per day; b.i.d.: two times per day; Aa: Aggregatibacter actinomycetemcomitans; Pg: Porphyromonas gingivalis.; *: included in the meta-analysis*

*Supplemental Table 4.* Description of studies included in the systematic review *(Continued)*.

| **Authors (year)**  **Country**  **Follow-up (Months)** | **Type of periodontitis & definition** | **Sampling strategy**  **Detection method** | **Groups: Sample size at baseline (drop outs); Age** | **Type of treatment** | **Antibiotic regimen, duration & timing of initiation** | **Maintenance regimen** | **Main results** |
| --- | --- | --- | --- | --- | --- | --- | --- |
| Guerrero et al.  (2014)*  United Kingdom  6 months | Generalized aggressive periodontitis.  AAP definition. | Four deepest sites in each quadrant.  PCR. | Test:  20(0); 31.3±5.2  Control:  21 (1); 31.7±5.1 | Test:  NSPT48+AMX+MTZ+chx  Control:  NSPT48+placebo+chx | AMX 500 mg t.i.d.  MTZ 500 mg t.i.d.  7 days.  Immediately after NSPT. | Recalls at 2 and 6 months. | Better eradication for *Pg* in test group but no difference for *Aa*.  Slight tendency to recolonization for *Pg* |
| Matarazzo et al.  (2008)*  Brazil  3 months | Chronic periodontitis.  ≥6 sites with PD between 5 and 7mm and CAL between 5 and 10mm. | 9 sites including 3 sites with: PD ≤ 3mm; PD 4-6mm; PD ≥7mm.  Checkerboard DNA-DNA hybridization. | Test:  15(1); 42.8±7.1  Control:  15 (0); 40.5±8.2 | Test:  NSPT+AMX+MTZ  Control:  NSPT+placebo | AMX 500 mg t.i.d.  MTZ 400 mg t.i.d.  14 days.  Immediately after first session of NSPT. | Unclear | No information regarding significant differences between groups.  *Aa* below detection threshold. |
| Mestnik et al.  (2010)*  Brazil  3 months | Generalized aggressive periodontitis.  ≥6 teeth including incisors and first molars with ≥1 site(s) with PD and CAL≥5mm and ≥6 other teeth with same conditions. | 9 sites including 3 sites with: PD ≤ 3mm; PD 4-6mm; PD ≥7mm.  Checkerboard DNA-DNA hybridization. | Test:  15 (0); 26.8±3.9  Control:  15 (0); 27.6±3.5 | Test:  NSPT+AMX+MTZ+chx  Control:  NSPT+placebo+chx | AMX 500 mg t.i.d.  MTZ 400 mg t.i.d.  14 days.  Immediately after first session of NSPT. | Unclear | Test group more effective for *Aa* and *Pg* at 3 months. |

*NSPT48: non-surgical periodontal therapy realized within 48 hours or less, except ultrasonic debridement only (USd); NSPT: non-surgical periodontal therapy realized in more than 48 hours; USd: 45 minutes ultrasonic debridement in one session; AMX: amoxicillin; MTZ: metronidazole; chx: chlorhexidine; t.i.d.: three times per day; b.i.d.: two times per day; Aa: Aggregatibacter actinomycetemcomitans; Pg: Porphyromonas gingivalis.; *: included in the meta-analysis*

*Supplemental Table 4.* Description of studies included in the systematic review *(Continued)*.

| **Authors (year)**  **Country**  **Follow-up (Months)** | **Type of periodontitis & definition** | **Sampling strategy**  **Detection method** | **Groups: Sample size at baseline (drop outs); Age** | **Type of treatment** | **Antibiotic regimen, duration & timing of initiation** | **Maintenance regimen** | **Main results** |
| --- | --- | --- | --- | --- | --- | --- | --- |
| Rooney et al.  (2002)*  United Kingdom  6 months | Chronic periodontitis.  ≥4 teeth in different quadrants with ≥1 site(s) with PD ≥6 plus BOP and/or suppuration | 1 site with PD ≥6mm in each quadrant.  Culture, enzymatic. | Test:  15 (1); <46.0  Control:  15 (1); <46.0 | Test:  NSPT+AMX+MTZ+chx  Control:  NSPT+placebo+chx | AMX 250 mg t.i.d.  MTZ 200 mg t.i.d.  7 days.  Immediately after NSPT. | Unclear | No sufficient data to explore microbiological differences from baseline and between groups. |
| Silva et al.  (2011)*  Brazil  3 months | Generalized chronic periodontitis.  ≥6 teeth with ≥1 site(s) with PD and CAL ≥5mm and 30% sites with CAL ≥4mm. | 9 sites including 3 sites with: PD ≤ 3mm; PD 4-6mm; PD ≥7mm.  Checkerboard DNA-DNA hybridization. | Test:  17 (0); 45.5±9.6  Control:  17 (0); 48.9±12.4 | Test:  NSPT+AMX+MTZ  Control:  NSPT+placebo | AMX 500 mg t.i.d.  MTZ 400 mg t.i.d.  14 days.  After first session of NSPT. | Unclear | No information regarding significant differences between groups. |
| Silva-Senem et al.  (2013)*  Brazil  12 months | Generalized aggressive periodontitis.  ≥4 teeth (3 other than central incisors and first molars) with ≥1 site(s) with PD≥6mm, CAL>5mm plus BOP. | 14 sites including 4 sites with PD ≤ 3mm; 5 sites with PD 4-6mm; 5 sites with PD ≥7mm.  Checkerboard DNA-DNA hybridization. | Test:  18 (2); 33.1±5.1  Control:  17 (2); 32.1±3.9 | Phase I: NSPT48+chx  Phase II (1 week after):  Test:  NSPT+AMX+MTZ+chx  Control:  NSPT+placebo+chx | AMX 500 mg t.i.d.  MTZ 250 mg t.i.d.  10 days.  I After first session of NSPT. | Recalls at 3, 6, and 9 months. | No significant reduction in *Aa* or *Pg* in both groups. |

*NSPT48: non-surgical periodontal therapy realized within 48 hours or less, except ultrasonic debridement only (USd); NSPT: non-surgical periodontal therapy realized in more than 48 hours; USd: 45 minutes ultrasonic debridement in one session; AMX: amoxicillin; MTZ: metronidazole; chx: chlorhexidine; t.i.d.: three times per day; b.i.d.: two times per day; Aa: Aggregatibacter actinomycetemcomitans; Pg: Porphyromonas gingivalis.; *: included in the meta-analysis*

*Supplemental Table 4.* Description of studies included in the systematic review *(Continued)*.

| **Authors (year)**  **Country**  **Follow-up (Months)** | **Type of periodontitis & definition** | **Sampling strategy**  **Detection method** | **Groups: Sample size at baseline (drop outs); Age** | **Type of treatment** | **Antibiotic regimen, duration & timing of initiation** | **Maintenance regimen** | **Main results** |
| --- | --- | --- | --- | --- | --- | --- | --- |
| Soares et al.  (2014)*  Brazil  12 months | Generalized chronic periodontitis.  ≥6 teeth with ≥1 site(s) with PD and CAL ≥5mm and 30% sites with CAL ≥4mm. | 9 sites including 3 sites with: PD ≤ 3mm; PD 4-6mm; PD ≥7mm.  Checkerboard DNA-DNA hybridization. | Test:  39 (5); 46.3±8.6  Control:  40 (6); 45.8±8.5 | Test:  NSPT+AMX+MTZ+chx  Control:  NSPT+placebo | AMX 500 mg t.i.d.  MTZ 400 mg t.i.d.  14 days.  Immediately after first session of NSPT. | Recalls at 3, 6, and 12 months. | No information regarding significant differences within groups.  Recolonization for *Pg* in control group at 12 months. |
| Viana Casarin et al.  (2012)  Brazil  6 months | Generalized aggressive periodontitis.  ≥8 teeth with ≥1 site(s) with PD≥5mm and ≥2 teeth with ≥1 site(s) with PD≥7mm | 1 site with PD =5mm and  1 site with PD ≥7mm.  PCR. | Test:  13 (1); 28.8±6.2  Control:  12 (0); 28.3±5.9 | Test:  USd+AMX+MTZ  Control:  USd+placebo | AMX 375 mg t.i.d.  MTZ 250 mg t.i.d.  7 days.  Immediately after USd. | Unclear | .No microbiological differences between groups.  Tendency towards recolonization in control group at 6 months. |
| Winkel et al.  (2001)*  Netherlands  6 months | Chronic periodontitis.  ≥1 site(s) in at least 3 of the 4 quadrants with PD>6mm and CAL≥3mm plus BOP and radiographic bone loss. | Deepest site with BOP in each quadrant.  Culture, enzymatic. | Test:  27 (4); 45.0 (32.0-63.0)  Control:  27 (1); 40.0 (28.0-55.0) | Phase I: NSPT  Phase II (6 weeks after):  Test:  NSPT48+AMX+MTZ  Control:  NSPT48+placebo | AMX 375 mg t.i.d.  MTZ 250 mg t.i.d.  7 days.  Initiation on the same day than phase II. | Unclear | No microbiological differences between groups.  Tendency towards recolonization in control group at 6 months. |

*NSPT48: non-surgical periodontal therapy realized within 48 hours or less, except ultrasonic debridement only (USd); NSPT: non-surgical periodontal therapy realized in more than 48 hours; USd: 45 minutes ultrasonic debridement in one session; AMX: amoxicillin; MTZ: metronidazole; chx: chlorhexidine; t.i.d.: three times per day; b.i.d.: two times per day; Aa: Aggregatibacter actinomycetemcomitans; Pg: Porphyromonas gingivalis.; *: included in the meta-analysis*

*Supplemental Table 4.* Description of studies included in the systematic review *(Continued)*.

| **Authors (year)**  **Country**  **Follow-up (Months)** | **Type of periodontitis & definition** | **Sampling strategy**  **Detection method** | **Groups: Sample size at baseline (drop outs); Age** | **Type of treatment** | **Antibiotic regimen, duration & timing of initiation** | **Maintenance regimen** | **Main results** |
| --- | --- | --- | --- | --- | --- | --- | --- |
| Xajigeorgiou et al.  (2006) *  Greece  6 months | Generalized aggressive periodontitis.  AAP definition. | 10 sites with PD >5mm.  Checkerboard DNA-DNA hybridization. | Test:  11 (1); 38·9±8·7  Control:  11 (0); 37.0±5.6 | Phase I: NSPT  Phase II (6 weeks after):  Test:  NSPT48+AMX+MTZ  Control:  USd | AMX 500 mg t.i.d.  MTZ 500 mg t.i.d.  7 days.  Initiation on the same day than phase II. | Unclear | No significant difference for *Aa* and *Pg* between groups. |
| Yek et al.  (2010)  Turkey  6 months | Generalized aggressive periodontitis.  ≥2 teeth in each quadrant with ≥1 site(s) with PD≥5mm and ≥3 teeth other than incisors and first molars with radiographic bone loss. | 3 deepest sites with at least PD ≥5mm.  PCR. | Test:  16 (4); 33.7±6.9  Control:  16 (0); 28.9±7.4 | Test:  NSPT+AMX+MTZ  Control:  NSPT | AMX 500 mg t.i.d.  MTZ 500 mg t.i.d.  7 days.  Immediately after first session of NSPT. | Recalls every month | No significant difference for *Aa* and *Pg* between groups.  Tendency towards recolonization at 6 months. |

*NSPT48: non-surgical periodontal therapy realized within 48 hours or less, except ultrasonic debridement only (USd); NSPT: non-surgical periodontal therapy realized in more than 48 hours; USd: 45 minutes ultrasonic debridement in one session; AMX: amoxicillin; MTZ: metronidazole; chx: chlorhexidine; t.i.d.: three times per day; b.i.d.: two times per day; Aa: Aggregatibacter actinomycetemcomitans; Pg: Porphyromonas gingivalis.; *: included in the meta-analysis*

1. [↑](#endnote-ref-2)
