# Supplementary material for: Detection of Porphyromonas gingivalis and Aggregatibacter actinomycetemcomitans after Systemic Administration of Amoxicillin Plus Metronidazole as an Adjunct to Non-surgical Periodontal Therapy: A Systematic Review and Meta-Analysis
Source: Front Microbiol. 2016 Aug 19;7:1277. doi: 10.3389/fmicb.2016.01277 (PMC4990718; doi:10.3389/fmicb.2016.01277)
Supplement: Supplementary Table 5 — Impact of mechanical treatment protocol, sampling methods, microbiological technology and type of periodontitis on the percentage of change in the detection of P. gingivalis and A. actinomycetemcomitans at 12-month follow-up. [file Table5.doc]

*Supplemental Table 5*. Impact of mechanical treatment protocol, sampling methods, microbiological technology and type of periodontitis on the percentage of change in the detection of *P. gingivalis* and *A.actinomycetemcomitans* at 12-month follow-up.

|  | **Mean difference (95% CI)** | **I^2^** | |  |
| --- | --- | --- | --- | --- |
| At 12-month follow-up, *A. actinomycetemcomitans* | | | | |
| Multiple sessions of NSPT (2,5) | -0.05 (-0.54-0.45) | | 71% | |
| Biofilm collection, sites with different probing depths (2,4,5) | -0.09 (-0.34-0.16) | | 49% | |
| Microbiological technology, Non-enzymatic technique (1,2,4,5) | 0.06 (-0.28-0.40) | | 80% | |
| At 12-month follow-up, *P. gingivalis* |  | |  | |
| Full-mouth disinfection (1,4) | 0.44 (-0.45-1.33) | | 96% | |
| Multiple sessions of NSPT (2,3,5) | 0.32 (0.14-0.49)^§^ | | 41% | |
| Biofilm collection, sites with different probing depths (2,3,4,5) | 0.22 (-0.04-0.47) | | 89% | |
| Microbiological technology, Non-enzymatic technique (1,2,3,4,5) | 0.34 (0.04-0.64)^*^ | | 93% | |
| Aggressive periodontitis (2,4) | 0.05 (-0.12-0.23) | | 54% | |
| Chronic periodontitis (1,3,5) | 0.41 (0.22-0.64)^§^ | | 80% | |

*Inverse variance method, random effects model.* ^*^p<0.05 ; ^§^p<0.01.*Full-mouth disinfection/multiple sessions : all non-surgical periodontal therapy (NSPT) realized within 48 hours or less / in more than 48 hours. PD= Probing depth. 1: Ehmke et al. 2005; 2: Mestnik et al. 2010; 3: Miranda et al. 2014; 4: Silva-Senem et al. 2013; 5: Soares et al. 2014.*

|  |  |  |  |
| --- | --- | --- | --- |
