# Supplementary figures and images for: Detection of Porphyromonas gingivalis and Aggregatibacter actinomycetemcomitans after Systemic Administration of Amoxicillin Plus Metronidazole as an Adjunct to Non-surgical Periodontal Therapy: A Systematic Review and Meta-Analysis
Source: Front Microbiol. 2016 Aug 19;7:1277. doi: 10.3389/fmicb.2016.01277 (PMC4990718; doi:10.3389/fmicb.2016.01277)

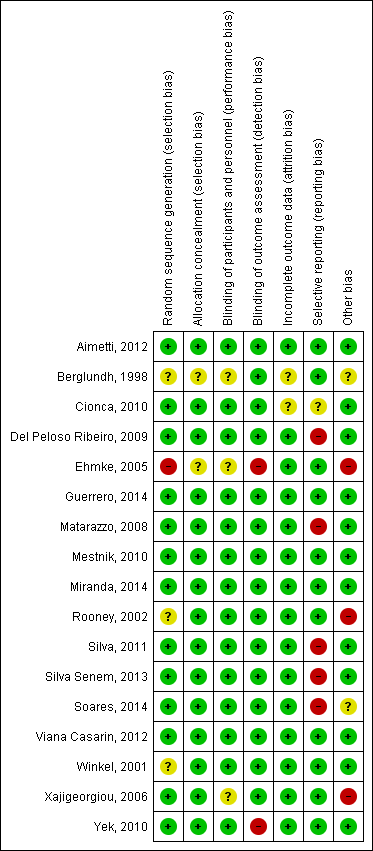

Supplement: Supplementary Figure 1 — Risk of bias assessment of included studies. [file Image1.PNG]
